# Using Artificial Intelligence (Watson for Oncology) for Treatment Recommendations Amongst Chinese Patients with Lung Cancer: Feasibility Study

**DOI:** 10.2196/11087

**Published:** 2018-09-25

**Authors:** Chaoyuan Liu, Xianling Liu, Fang Wu, Mingxuan Xie, Yeqian Feng, Chunhong Hu

**Affiliations:** 1 Department of Oncology The Second Xiangya Hospital Central South University Changsha, Hunan China; 2 Department of Geriatric Medicine Xiangya Hospital Central South University Changsha, Hunan China

**Keywords:** Watson for Oncology, artificial intelligence, lung neoplasms, comparative study, interdisciplinary communication

## Abstract

**Background:**

Artificial intelligence (AI) is developing quickly in the medical field and can benefit both medical staff and patients. The clinical decision support system Watson for Oncology (WFO) is an outstanding representative AI in the medical field, and it can provide to cancer patients prompt treatment recommendations comparable with ones made by expert oncologists. WFO is increasingly being used in China, but limited reports on whether WFO is suitable for Chinese patients, especially patients with lung cancer, exist. Here, we report a retrospective study based on the consistency between the lung cancer treatment recommendations made for the same patient by WFO and by the multidisciplinary team at our center.

**Objective:**

The aim of this study was to explore the feasibility of using WFO for lung cancer cases in China and to ascertain ways to make WFO more suitable for Chinese patients with lung cancer.

**Methods:**

We selected all lung cancer patients who were hospitalized and received antitumor treatment for the first time at the Second Xiangya Hospital Cancer Center from September to December 2017 (N=182). WFO made treatment recommendations for all supported cases (n=149). If the actual therapeutic regimen (administered by our multidisciplinary team) was recommended or for consideration according to WFO, we defined the recommendations as consistent; if the actual therapeutic regimen was not recommended by WFO or if WFO did not provide the same treatment option, we defined the recommendations as inconsistent. Blinded second round reviews were performed by our multidisciplinary team to reassess the incongruent cases.

**Results:**

WFO did not support 18.1% (33/182) of recommendations among all cases. Of the 149 supported cases, 65.8% (98/149) received recommendations that were consistent with the recommendations of our team. Logistic regression analysis showed that pathological type and staging had significant effects on consistency (*P*=.004, odds ratio [OR] 0.09, 95% CI 0.02-0.45 and *P*<.001, OR 9.5, 95% CI 3.4-26.1, respectively). Age, gender, and presence of epidermal growth factor receptor gene mutations had no effect on consistency. In 82% (42/51) of the inconsistent cases, our team administered two China-specific treatments, which were different from the recommendations made by WFO but led to excellent outcomes.

**Conclusions:**

In China, most of the treatment recommendations of WFO are consistent with the recommendations of the expert group, although a relatively high proportion of cases are still not supported by WFO. Therefore, WFO cannot currently replace oncologists. WFO can improve the efficiency of clinical work by providing assistance to doctors, but it needs to learn the regional characteristics of patients to improve its assistive ability.

## Introduction

### China's Medical Contradictions

The contradiction between high-quality medical resources and people’s medical needs is becoming increasingly prominent. These contradictions have led to many medical conflicts in China, and they have even attracted the attention of the international community [1-4]. There are several reasons for such contradictions. First, due to the severe shortage of government investment in health-related areas resulting in a severe lack of medical resources, Chinese doctors in primary health services are usually overworked [4-6]. There are only 1.2 physicians for every 1000 individuals in China, compared with almost 2.8 physicians for every 1000 individuals in America and other developed countries [4,6]. Over 32% of physicians work more than 60 hours per week in China [5], 94% of Chinese doctors have reported being in a poor condition every day after work, and 48% of doctors reported feeling very tired [6]. Thus, doctors in China may not have enough time or energy to ensure that their knowledge is at par with the current developments in the field of cancer. On the other hand, medical data, papers, and guidelines in tumor-related fields are rapidly growing, whereas the time doctors can dedicate to learning is limited. In October 2017, the Food and Drug Administration of the United States approved 69 drugs for the treatment of breast cancer alone, and the National Comprehensive Cancer Network guide for lung cancer was updated 9 times in 2017. Studies have shown that oncologists spend only 4.6 hours per week acquiring professional knowledge [7]. However, it is more urgent for specialists to obtain timely knowledge of evidence-based medicine than for doctors in other clinical disciplines to support individualized treatment plans for patients. Even oncologists who are experts in a specialized field cannot master all available knowledge; furthermore, doctors at the primary level are tasked with tackling numerous tumor types. Second, the distribution of medical resources is not balanced. The same patient may receive different treatment recommendations across different hospitals, and even within the same hospital, different doctors may offer different treatment options. A previous study noted that the Chinese government needs to strengthen its investment in medical professions among young physicians [8]. A tool that can help Chinese doctors to quickly provide accurate treatment recommendations or help doctors to learn new developments in the field more efficiently is urgently needed.

### A Brief Introduction of Watson for Oncology and Its Popularity in China

IBM’s Watson for Oncology (WFO, IBM Corporation, United States), which has been previously described by Somashekhar [9], is a clinical decision-support system for oncology therapy selection. In short, WFO has stored and indexed literature, protocols, and patient charts; has learned from test cases and experts from Memorial Sloan Kettering Cancer Center (MSKCC); and can apply computational reasoning approaches to speciﬁc cases [9]. All the information input is verified by the top oncologists at MSKCC. Moreover, WFO data are updated to the latest cutting-edge information every 1 to 2 months. It is described in the manual that WFO does not support certain cases. When we input a case that is not supported by WFO, the WFO system does not process the case, and no recommendation is returned. For lung cancer, the unsupported cases include patients with isolated metastatic tumors and patients with driver mutations whose cancer progresses during metastatic therapy, etc. For supported cases, the treatment recommendations provided by WFO are categorized into 3 groups: *recommended*, which represents a treatment supported by strong evidence; *for consideration*, which represents a potentially suitable alternative; and *not recommended*, which represents a treatment with contraindications or strong evidence against its use [9]. We speculated that WFO might be a viable option to solve problems in the health care setting in China. WFO requires only the data of a case to be input, and within 1 min, it outputs the most standard treatment approach recommended for the specified case with highly consistent evidence [9]. In fact, the use of WFO is becoming increasingly prevalent in China: WFO was introduced to China in March 2017 and currently serves more than 70 medical institutions nationwide above the city level and more than 10,000 patients [10].

### Unresolved Issues

With the growing popularity of WFO, which was developed in the United States, many doctors and medical institutions in China have questioned to what extent WFO is suitable for Chinese cancer patients. Patients with cancer also question whether they can receive treatment recommendations from WFO. The problem is two-fold. On one hand, what percentage of all cancer cases is unsupported by WFO? For various reasons, WFO does not support some cases. If the proportion of unsupported cases is very large (such as greater than 50%), although the concordance of supported cases between WFO and the local multidisciplinary team (MDT) is high, the total application value of WFO would be questionable. On the other hand, among the supported cases, how consistent are the recommendations from WFO with those from the MDT? Furthermore, how can we make WFO more suitable for Chinese people? Few reports have considered these questions [11-13]. Existing studies in China all pertain to the consistency of the recommendations from WFO with those from the MDT for cancer patients; however, their sample sizes have been small, especially for studies on lung cancer [11-13], which has the highest morbidity and mortality among malignant tumors in the world or in China [14]. Moreover, none of the previous studies referred to unsupported cases or how to improve the performance of WFO [11-13].

To address these unresolved issues, we conducted a retrospective study on lung cancer in our center and compared the treatment recommendations output by WFO and the actual treatment provided by the MDT in our hospital. Here, we report the specific details and results of this study.

## Methods

### Introduction of Our Center and the Multidisciplinary Team

The Second Xiangya Hospital is one of the teaching hospitals and clinical schools affiliated with Central South University in China. It is one of only 10 key medical colleges cofounded by the Ministry of Education and Ministry of Health in China and is one of the top 100 tertiary care hospitals in China [15]. Our center is part of the Second Xiangya Hospital and serves approximately 30,000 cancer patients each year, and it is the largest and best comprehensive cancer treatment and research center in Hunan Province [16].

The MDT of our hospital is composed of oncologists, radiation oncologists, surgeons, radiologists, pathologists, and palliative care specialists. When a patient is hospitalized, the physician in charge collects all the necessary medical data and assembles the relevant medical experts to form the MDT. After discussion, the MDT forms and implements a comprehensive regimen.

### Patient Selection

The study protocol was approved by the medical ethics committee of the Second Xiangya Hospital (ID: 2017-S104). Data were collected on patients who received antitumor treatment at our center. The inclusion criteria for this study were as follows: (1) inpatients at our center; (2) lung cancer patients; (3) admission between September 2017 and December 2017; and (4) received antitumor treatment for the first time. The exclusion criteria were as follows: (1) those who received only examinations and did not receive any antitumor treatment and (2) those who had previously received antitumor treatment. A total of 182 cases were included in this study. The pathological types included squamous carcinoma, adenocarcinoma, adenosquamous carcinoma, large-cell carcinoma, and small-cell carcinoma. The treatment received included postoperative adjuvant therapy, definitive therapy, and best supportive therapy. A total of 33 cases were not supported by WFO, and thus, the remaining 149 patients were included in our comparison study.

### Operating Procedure and Evaluation of Consistency

**Figure 1:**
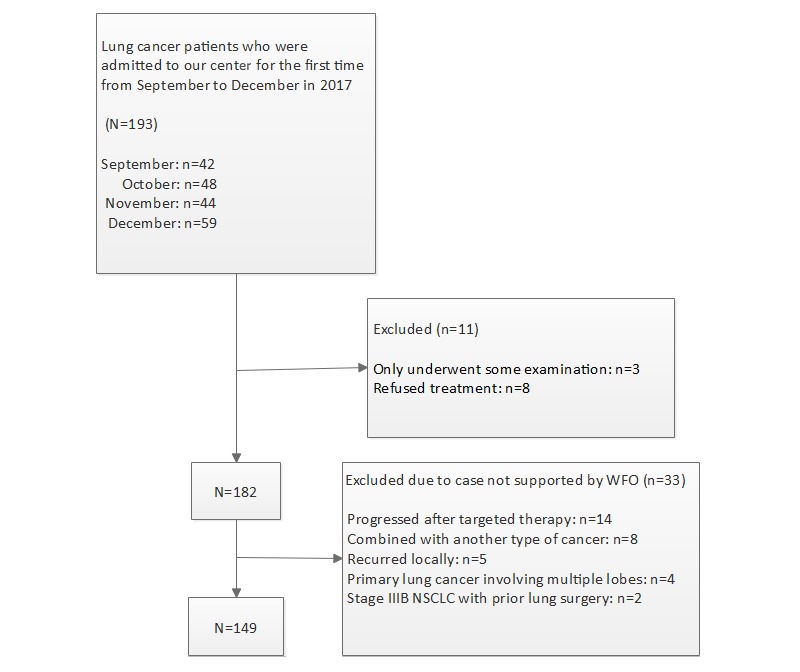
Flow diagram of the patient selection process. NSCLC: nonsmall cell lung cancer; WFO: Watson for Oncology.

**Figure 2:**
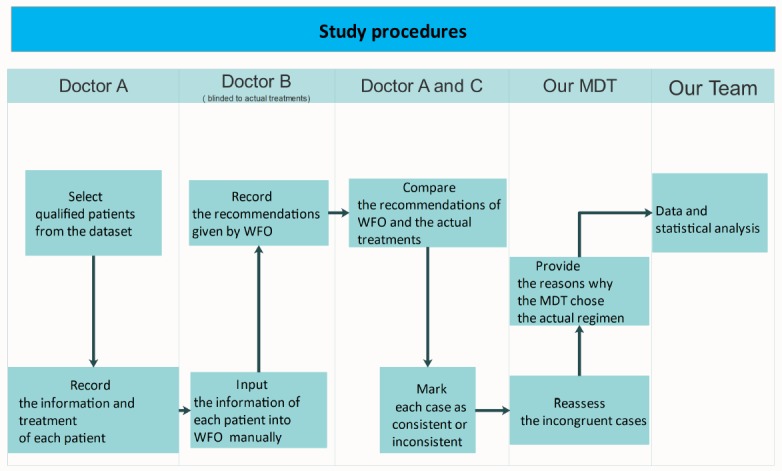
Flow diagram of the study procedure. MDT: multidisciplinary team; WFO: Watson for Oncology.

A flow diagram of the patient selection process is shown in Figure 1, and the study procedures are shown in Figure 2.

Patient information and specific treatments were collected from the electronic medical record system of our hospital (see Figure 1); a senior physician who was blinded to the actual treatments manually input the patient information into WFO (IBM Watson 17.1) and recorded the recommendation made by WFO. Two other doctors compared the recommendation from WFO and the actual treatment. If our actual therapeutic regimen was deemed *recommended* or *for consideration* by WFO, we defined the outcome as consistent, and if the actual treatment was deemed *not recommended* or not included in the recommendation by WFO, we defined the outcome as inconsistent. The team of specialists at our center reassessed the incongruent cases and provided their reasons for choosing the actual regimens.

### Data Analysis and Statistics

We used Microsoft Excel and SPSS 23.0 to describe the data and to perform the statistical analysis. A logistic regression model was estimated with odds ratios and 95% CIs.

## Results

### Characteristics of the Included Lung Cancer Cases

Demographic characteristics of the hospitalized lung cancer patients are shown in Table 1. Overall, 18.1% (33/182) of our patients’ cases were not supported by WFO. Among the supported cases, nonsmall cell lung cancer (NSCLC) accounted for 84.6% (126/149) and small-cell lung cancer (SCLC) accounted for 15.4% (23/149); these proportions were largely consistent with the pathological distribution of lung cancer worldwide [8]. The median age of our patients was 60 years, of whom 83.2% (124/149) were males and 16.8% (25/149) were females. Adenocarcinoma with the wild-type epidermal growth factor receptor (EGFR) gene accounted for 69% (42/61) of all patients, and phase III and IV disease accounted for 81.2% (121/149) of all patients.

### Consistency of Supported Cases and the Influencing Factors

**Figure 3:**
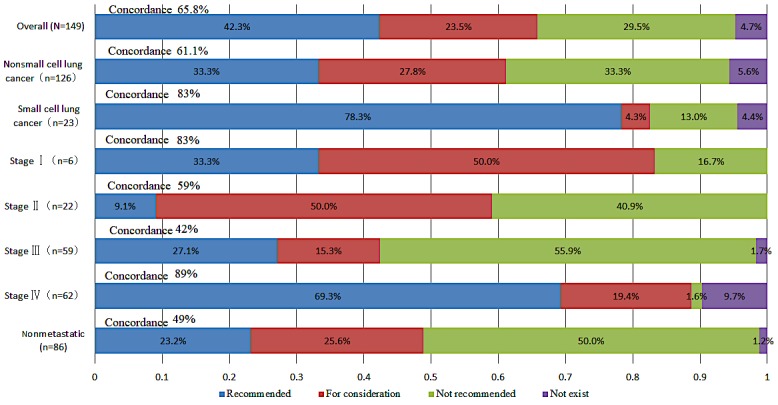
Overall treatment concordance between Watson for Oncology and the multidisciplinary team, divided by stage and pathology category.

After our team of specialists reassessed the incongruent patients, there was no change to the primary concordance. The general consistency was 65.8% (98/149, shown in Table 2), and the consistency for nonmetastatic cases was 49% (42/86). For metastatic cases, the consistency was 87% (55/63); for NSCLC, the consistency was 61.1% (77/126); and for SCLC, the consistency was 83% (19/23, shown in Figure 3). The logistic regression analysis showed that age (which was divided into two groups: >60 years old and ≤60 years old, *P*=.45), gender (*P*=.30), and the presence of EGFR gene mutation (*P*=.90) had no effect on consistency (shown in Table 3). The following factors had significant effects on consistency: pathological type (*P*=.004), with the consistency in SCLC cases being 83% (19/23) and consistency in NSCLC cases being 61.1% (77/126); and stage (*P*<.001), including stage I (83%, 5/6), stage II (59%, 13/22), stage III (42%, 25/59), and stage IV (89%, 55/62). There were 2 major reasons accounting for 80% (41/51) of the inconsistent cases. First, we adopted sequential chemoradiation instead of concurrent chemoradiation. Second, we adopted icotinib and Endostar instead of the other first-generation epidermal growth factor receptor tyrosine kinase inhibitor (EGFR-TKI) and bevacizumab. If WFO was able to output these two alternative treatments as *recommended* or *for consideration*, the overall consistency could be elevated from 65.8% (98/149) to 93.2% (139/149).

**Table 1:** Characteristics of lung cancer cases (N=149).

Characteristics		Statistics
**Sex, n (%)**		
	Male	124 (83.2)
	Female	25 (16.8)
Median age in years (range)		60 (26-83)
**Pathology, n (%)**		
	Squamous carcinoma	61 (40.9)
	Adenocarcinoma	61 (40.9)
	Adenosquamous carcinoma	3 (2.1)
	Small-cell carcinoma	23 (15.4)
	Large-cell carcinoma	1 (0.7)
**Stage, n (%)**		
	I	6 (4.0)
	II	22 (14.8)
	III	59 (39.6)
	IV	62 (41.6)
**Epidermal growth factor receptor (EGFR) gene mutation status, n (%)**		
	EGFR mutation	8 (5.4)
	Wild-type EGFR	44 (29.5)
	Unknown	97 (65.1)

**Table 2:** Multidisciplinary team and Watson for Oncology recommendations after the initial reviews (N=149).

Reviews of lung cancer cases	Recommendations	Availability	Total
Concordant cases, n (%)	63 (42.3)^a^	35 (23.5)^b^	98 (65.8)
Nonconcordant cases, n (%)	44 (29.5)^c^	7 (4.7)^d^	51 (34.2)

^a^Recommended.

^b^For consideration.

^c^Not recommended.

^d^Not available.

**Table 3:** Logistic regression model of concordance between Watson for Oncology and the multidisciplinary team.

Characteristics		Odds ratio (95% CI)	*P* value
Age (≤60 years, >60 years)		0.72 (0.31-1.70)	.45
Gender		0.54 (0.17-1.74)	.30
Pathology (NSCLC^a^ and SCLC^b^)		0.09 (0.02-0.45)	.004
**Stages (stage I reference)**			
	Stage II	1 (0.09-10.7)	>.99
	Stage III	3.51 (1.03-12.0)	.05
	Stage IV	9.5 (3.4-26.1)	<.001
**EGFR^c^gene mutation (reference)**			
	Wild-type EGFR	0.91 (0.16-5.26)	.91
	Nonmeasured	0.32 (0.12-0.86)	.02

^a^NSCLC: nonsmall cell lung cancer.

^b^SCLC: small-cell lung cancer

^c^EGFR: epidermal growth factor receptor.

## Discussion

### Disadvantages of Applying Watson for Oncology in Chinese Lung Cancer Cases

This retrospective study suggests that WFO requires improvement before application in Chinese lung cancer cases. As mentioned in the manual, WFO does not support certain cases. We suspect the possible reasons are that these cases are complicated and cannot be addressed by the current technology of WFO. In this study, WFO did not support 18.1% (33/182) of the cases, of which 42% (14/33) progressed after targeted therapy. There is a large difference in the EGFR gene mutation phenotype of lung cancer in China compared with that in Western countries. The EGFR mutation rate of lung cancer in European and American countries is approximately 15%, whereas the probability of this mutation is 50% or more in China [17,18]. The treatment consistency was 65.8% (98/149), which was much lower than the treatment consistency of 96.4% reported in an abstract at the 2017 American Society of Clinical Oncology Annual Meeting [19]. There are several reasons for this discrepancy. First, WFO recommends concurrent chemoradiation, whereas China performs sequential chemoradiation (67%, 34/51); the physique of Chinese patients is usually weaker than that of Western patients, and thus, Chinese patients often cannot tolerate concurrent chemoradiation. Second, China uses the drugs Icotinib and Endostar instead of the other first-generation drugs EGFR-TKI and bevacizumab, respectively (14%, 7/51). Icotinib and Endostar [20-23] are primary research drugs in China, and studies have shown that they are as effective as the other first-generation drugs EGFR-TKI and bevacizumab in patients with lung cancer in China [24,25]. If WFO was able to output these 2 alternative treatments as *recommended* or *for consideration*, the overall consistency could be elevated from 65.8% to 93.2%. Third, some drugs are not available in the Chinese market, such as immune-targeted therapy drugs involving programmed death-1 and programmed death-ligand 1 antibodies. Patient preferences, prices, and medical insurance are also taken into consideration, and they ultimately affect the inconsistency. Moreover, WFO does not take some coexisting diseases into consideration. For example, in our study, one patient who was diagnosed with stage III squamous cell lung carcinoma also suffered from active tuberculosis. Receiving the standard chemoradiotherapy recommended by WFO may cause tuberculosis to spread rapidly, which may result in rapid death; therefore, our treatment strategy was the prescription of oral antituberculosis drugs before chemoradiotherapy. If this individualized information could be incorporated into WFO, the consistency of recommendations would be significantly improved.

### Advantages of Watson for Oncology

Although the consistency of recommendation from WFO with those from the MDT in this study was not as high as expected, and a portion of cases was not supported, WFO still has tremendous value. First, WFO provides evidence to support its recommendations, and this evidence is presented according to the credibility based on the related literature such as studies and related data [9]. The doctor could examine the related evidence to judge whether it applies to the current case. When the doctor selects a treatment, WFO also provides the survival rate, incidence of adverse effects, and other information related to the selected treatment to help doctors assess the curative effect of the regimen and risk as a whole. Second, WFO can markedly shorten the time that junior doctors must spend consulting relevant literature, thus improving their ability to make accurate diagnoses and treatment recommendations in a short time. Third, regarding patients, WFO can eliminate the time wasted by visiting various top hospitals and help patients obtain the best treatment as soon as possible. Fourth, WFO can solve the problem of doctor-patient trust. In contemporary China, for a variety of reasons such as funding shortage, excessive market-oriented operation, limitations in health insurance reimbursement amounts, and abundant nonneutral media coverage of health events, patients’ distrust of doctors is growing [26-28]; in addition, patients often suspect doctors of overtreatment. Unlike a local expert, who may provide some recommendations based on his or her own interests, WFO does not have personal preferences; therefore, WFO may be viewed as fair, and it can gain the trust of patients. As a result, cancer patients will not have to visit multiple experts to find a treatment regimen that they consider to be fair.

In conclusion, WFO can provide fast and accurate treatment recommendations to the majority of Chinese lung cancer patients, and it will play an important role in reducing the workload of doctors and in teaching young physicians. Moreover, WFO will standardize the treatment of lung cancer nationwide and enhance trust between doctors and patients, which is of significance to developing countries such as China, whose medical growth is unbalanced.

### Importance of Our Study

This study is very important. With the AI boom, this study provides a basis for medical institutions, medical staff, and cancer patients to obtain a better understanding of WFO and rational decision making. Today, with the rapid development of AI, some people blindly follow the recommendations of AI technologies. AI can be perceived to be able to do anything, and it has been postulated that AI could replace doctors in the future. However, our research shows that this change is impossible. Medicine is not just a science; medicine involves additional aspects such as social and psychological factors. Doctors must consider individualized measures for different patients, even those with the same diagnosis. In using WFO, we can see that WFO needs oncologists to confirm whether the patient can undergo a radical operation or radiation therapy, whether the patient can tolerate an operation or radiation, and whether there is any emergency; hence, the program can continue only if this information can be provided. After WFO provides the treatment recommendations, we must choose the most suitable treatment plan according to the patient’s physical and mental state, economic situation, complications, and willingness to accept treatment. It is important that WFO only stores existing knowledge and that it provides patients the best treatment recommendation that is currently available worldwide. Nonetheless, scientists must continue their research efforts to further medical advances while WFO acts as an assistant to doctors.

There is limited research on WFO [9,11-13]; we were the first to report on unsupported cases, and the sample size of this lung cancer study was the largest among all lung cancer studies performed in China. We not only reported the consistency of the recommendations from WFO with those from our MDT but also analyzed the influencing factors and provided some suggestions for the improvement of WFO to better suit Chinese patients.

### Limitations of Our Study

There are several limitations in our study. First, this study is a retrospective observational study with no controls. Second, the distribution is imbalanced among the groups of patients: fewer patients were stage I, which may be because we included hospitalized patients, and stage I patients are required to undergo observation only after surgery, and they do not need to be hospitalized for further treatment. There were also fewer women than men, as female patients mostly have adenocarcinoma pathology with EGFR mutations; most of these patients need to take only oral targeted drugs outside the hospital and do not need to be hospitalized. Third, the bias of input data from experts may lead to different treatment recommendations.

### Methods to Improve Watson for Oncology

AI generally has a much stronger memorization ability than the human brain, and it can quickly collect and sort the information that it stores, thus yielding accurate conclusions faster than humans, such as for applications in diagnostic radiology and pathology imaging systems [29,30]. However, to adapt to the real-world setting of China, WFO must be significantly improved. Following the acquisition of ethics approval, the medical data of patients must be standardized and shared nationwide, and the follow-up system must be improved to obtain complete information about the patients. In other words, we should build a unique medical data repository for China to be studied by WFO. These data should be incorporated with international guidelines and health care systems to allow WFO to reach its full potential to serve Chinese patients.

### Conclusions

WFO is currently not a substitute for oncologists. WFO is a good assistant for Chinese doctors and a good teacher for young physicians; it also helps to standardize the treatment of lung cancer nationwide. However, it needs to learn the local characteristics of patients to better serve Chinese lung cancer patients.

## Abbreviations

AI: artificial intelligence
EGFR: epidermal growth factor receptor
EGFR-TKI: epidermal growth factor receptor-tyrosine kinase inhibitor
MSKCC: Memorial Sloan Kettering Cancer Center
MDT: multidisciplinary team
NSCLC: nonsmall cell lung cancer
SCLC: small-cell lung cancer
WFO: Watson for Oncology

## References

[1] Jie L (2012). New generations of Chinese doctors face crisis. Lancet.

[2] Wang XQ, Wang XT, Zheng JJ (2012). How to end violence against doctors in China. Lancet.

[3] Yang T, Zhang H, Shen F, Li JW, Wu MC (2013). Appeal from Chinese doctors to end violence. Lancet.

[4] Zhao L, Zhang XY, Bai GY, Wang YG (2014). Violence against doctors in China. Lancet.

[5] Wong CW, Chan YH, Cheng YH, Lam CS (2016). Is overwork a precipitant factor of idiopathic ventricular fibrillation?. Int J Cardiol.

[6] Shan HP, Yang XH, Zhan XL, Feng CC, Li YQ, Guo LL, Jin HM (2017). Overwork is a silent killer of Chinese doctors: a review of Karoshi in China 2013-2015. Public Health.

[7] Woolhandler S, Himmelstein DU (2014). Administrative work consumes one-sixth of U.S. physicians' working hours and lowers their career satisfaction. Int J Health Serv.

[8] Lin Y, Yin S, Lai S, Tang J, Huang J, Du L (2016). The influence factors of medical professionalism: a stratified-random sampling study based on the physicians and patients in ambulatory care clinics of Chengdu, China. Medicine (Baltimore).

[9] Somashekhar SP, Sepúlveda MJ, Puglielli S, Norden AD, Shortliffe EH, Rohit Kumar C, Rauthan A, Arun Kumar N, Patil P, Rhee K, Ramya Y (2018). Watson for Oncology and breast cancer treatment recommendations: agreement with an expert multidisciplinary tumor board. Ann Oncol.

[10] (2018). Cn-healthcare.

[11] Zhang XC, Zhou N, Zhang CT, Lv HY, Li TJ, Zhu JJ, Jiang M, Hou HL, Liu D, Li AQ, Liu G, Zhang GQ (2017). 544P Concordance study between IBM Watson for Oncology (WFO) and clinical practice for breast and lung cancer patients in China. Ann Oncol.

[12] Zhou N, Lv H, Zhang C, Li T, Zhu J, Jiang M, Hou H, Liu D, Li A, Liu G, Liu K, Zhang G, Zhang X (2017). P1.01-069 Clinical experience with IBM Watson for Oncology (WFO) cognitive system for lung cancer treatment in China. J Thorac Oncol.

[13] Yue L, Yang L (2017). Clinical experience with IBM Watson for Oncology (WFO) for multiple types of cancer patients in China. Ann Oncol.

[14] Siegel RL, Miller KD, Jemal A (2018). Cancer statistics, 2018. CA Cancer J Clin.

[15] (2018). Xyeyy.

[16] (2018). Xyeyy.

[17] Li T, Kung HJ, Mack PC, Gandara DR (2013). Genotyping and genomic profiling of non-small-cell lung cancer: implications for current and future therapies. J Clin Oncol.

[18] Zhou C (2014). Lung cancer molecular epidemiology in China: recent trends. Transl Lung Cancer Res.

[19] Suwanvecho S, Suwanrusme H, Sangtian M, Norden AD, Urman A, Hicks A, Dankwa-Mullan I, Rhee K, Kiatikajornthada N (2017). Concordance assessment of a cognitive computing system in Thailand. J Clin Oncol.

[20] Lu S, Li L, Luo Y, Zhang L, Wu G, Chen Z, Huang C, Guo S, Zhang Y, Song X, Yu Y, Zhou C, Li W, Liao M, Li B, Xu L, Chen P, Hu C, Hu C (2015). A multicenter, open-label, randomized phase II controlled study of rh-endostatin (Endostar) in combination with chemotherapy in previously untreated extensive-stage small-cell lung cancer. J Thorac Oncol.

[21] Sun Y, Wang JW, Liu YY, Yu QT, Zhang YP, Li K, Xu LY, Luo SX, Qin FZ, Chen ZT, Liu WC, Zhou QH, Chen Q, Nan KJ, Liu XQ, Liu W, Liang HJ, Lu HS, Wang XW, Wang JJ, Song SP, Tu YR, Zhou JM, Li WL, Yao C, Endostar Phase III NSCLC Study Group (2013). Long-term results of a randomized, double-blind, and placebo-controlled phase III trial: Endostar (rh-endostatin) versus placebo in combination with vinorelbine and cisplatin in advanced non-small cell lung cancer. Thorac Cancer.

[22] Wang J, Gu LJ, Fu CX, Cao Z, Chen QY (2014). Endostar combined with chemotherapy compared with chemotherapy alone in the treatment of nonsmall lung carcinoma: a meta-analysis based on Chinese patients. Indian J Cancer.

[23] Han B, Xiu Q, Wang H, Shen J, Gu A, Luo Y, Bai C, Guo S, Liu W, Zhuang Z, Zhang Y, Zhao Y, Jiang L, Shi C, Jin B, Zhou J, Jin X (2011). [A multicenter, randomized, double-blind, placebo-controlled safety study to evaluate the clinical effects and quality of life of paclitaxel-carboplatin (PC) alone or combined with endostar for advanced non-small cell lung cancer (NSCLC)]. Zhonghua Zhong Liu Za Zhi.

[24] Grigoriu B, Berghmans T, Meert AP (2015). Management of EGFR mutated nonsmall cell lung carcinoma patients. Eur Respir J.

[25] Shi Y, Zhang L, Liu X, Zhou C, Zhang L, Zhang S, Wang D, Li Q, Qin S, Hu C, Zhang Y, Chen J, Cheng Y, Feng J, Zhang H, Song Y, Wu YL, Xu N, Zhou J, Luo R, Bai C, Jin Y, Liu W, Wei Z, Tan F, Wang Y, Ding L, Dai H, Jiao S, Wang J, Liang L, Zhang W, Sun Y (2013). Icotinib versus gefitinib in previously treated advanced non-small-cell lung cancer (ICOGEN): a randomised, double-blind phase 3 non-inferiority trial. Lancet Oncol.

[26] Zhou M, Zhao L, Campy KS, Wang S (2017). Changing of China׳s health policy and doctor&ndash;patient relationship: 1949&ndash;2016. Health Policy Technol.

[27] Zhou P, Grady SC (2016). Three modes of power operation: understanding doctor-patient conflicts in China's hospital therapeutic landscapes. Health Place.

[28] Chan CS (2018). Mistrust of physicians in China: society, institution, and interaction as root causes. Dev World Bioeth.

[29] Fazal MI, Patel ME, Tye J, Gupta Y (2018). The past, present and future role of artificial intelligence in imaging. Eur J Radiol.

[30] Thompson RF, Valdes G, Fuller CD, Carpenter CM, Morin O, Aneja S, Lindsay WD, Aerts HJ, Agrimson B, Deville C, Rosenthal SA, Yu JB, Thomas CR (2018). Artificial intelligence in radiation oncology: a specialty-wide disruptive transformation?. Radiother Oncol.

